# Investigation of evolutionary dynamics for drug resistance in 3D spheroid model system using cellular barcoding technology

**DOI:** 10.1371/journal.pone.0291942

**Published:** 2023-09-26

**Authors:** Gizem Damla Yalcin, Kubra Celikbas Yilmaz, Tugce Dilber, Ahmet Acar

**Affiliations:** Department of Biological Sciences, Middle East Technical University, Çankaya, Ankara, Turkey; National Institute of Cancer Research, TAIWAN

## Abstract

Complex evolutionary dynamics governing the drug resistance is one of the major challenges in cancer treatment. Understanding these mechanisms requires a sequencing technology with higher resolution to delineate whether pre-existing or de novo drug mechanisms are behind the drug resistance. Combining this technology with clinically very relevant model system, namely 3D spheroids, better mimicking tumorigenesis and drug resistance have so far been lacking. Thus, we sought to establish dabrafenib and irinotecan resistant derivatives of barcoded 3D spheroids with the ultimate aim to quantify the selection-induced clonal dynamics and identify the genomic determinants in this model system. We found that dabrafenib and irinotecan induced drug resistance in 3D-HT-29 and 3D-HCT-116 spheroids are mediated by pre-existing and de novo resistant barcodes, indicating the presence of polyclonal drug resistance in this system. Moreover, whole-exome sequencing analysis found chromosomal gains and mutations associated with dabrafenib and irinotecan resistance in 3D-HT-29 and 3D-HCT-116 spheroids. Last, we show that dabrafenib and irinotecan resistance are also mediated by multiple drug resistance by detection of upregulation of the drug efflux pumps, *ABCB1* and *ABCG2*, in our spheroid model system. Overall, we present the quantification of drug resistance and evolutionary dynamics in spheroids for the first time using cellular barcoding technology and the underlying genomic determinants of the drug resistance in our model system.

## Introduction

Three-dimensional (3D) cell culture models, which provide a more physiologically relevant environment than typical two-dimensional (2D) cell culture, have emerged as significant tools in cancer research [1–3]. In recent years, 3D spheroids have received an increased interest among the many 3D culture systems [4, 5]. 3D spheroids are multicellular aggregates that more precisely mirror the structural and functional properties of tissues, providing useful models for investigating cancer progression and drug resistance [6]. Indeed, 3D cell culture models enable cells to maintain physiological characteristics such as cell polarization, differentiation, and cell-to-cell signalling, which are critical for understanding the processes in cancer progression and drug resistance [7, 8].

Because of their capacity to recapitulate the complex cellular interactions present in tumours, 3D spheroids have emerged as critical models in cancer research [9]. For example, 3D spheroids can more closely resemble the architecture of actual tumours than typical 2D cell cultures [10]. They have proven to be comparable to the tumour microenvironment in vivo with the heterogeneous cell populations that are arranged in distinct layers [11]. Similar to *in vivo* solid tumours, cells in the spheroids are actively proliferating on the outer layer (proliferative zone), quiescent on the inner layer (quiescent zone), and necrotic in the centre (necrotic zone) of the spheroids [6]. As a result of the hypoxia present and lack of drug penetrance in the inner zone of spheroids, elevated levels of Hypoxia-Inducible Factor 1 (HIF-1) and P-glycoprotein have been linked to drug resistance in a number of cancer cell models [12–14].

Drug resistance is arguably one of the biggest challenges to effective treatment solutions [15, 16]. Depending on the genomic background of the patient’s tumour, small-molecule inhibitors can be administered [17]. For example, dabrafenib targets mutated BRAF proteins and is used in the treatment of CRC and melanoma patients harbouring BRAF V600E mutations [18, 19]. In addition, chemotherapeutic agents such as oxaliplatin, irinotecan, SN-38, 5-fluororacil, and capecitabine are frequently used in the treatment of CRC patients [20, 21]. Despite the initial effects of these treatment strategies in shrinking the tumors, resistance invariably develops either through the expansion of pre-existing resistant subpopulations or the de novo emergence of resistant populations [22–26]. Understanding the dynamics of drug-resistant subpopulations under the selective forces of targeted or chemotherapeutic drugs can provide critical insights into developing more effective therapeutic solutions for overcoming drug resistance [27].

Tumours frequently demonstrate genomic heterogeneity, with various (epi)genetic changes found in different subclones [28, 29]. The use of DNA barcoding can assist in tracking the evolution and dynamics of tumour heterogeneity [30–32]. Through this technology, the presence and quantity of different subclones inside a tumour can be identified via barcoding the genome, yielding insights about clonal evolution, metastatic potential, and response to therapy [33–35]. In comparison to conventional NGS methods, cellular barcoding technology provides in-depth resolution to detect and track clonal evolution in in vitro and in vivo model systems, with the ultimate goal of understanding the underlying mechanisms of cancer progression in growing populations and drug-induced selection forces [36, 37]. For example, a few studies, including ours, have recently revealed the presence of pre-existing and de novo drug resistance mechanisms in an experimental evolution context using cellular barcoding technology [32, 38, 39].

In this study, we present a 3D spheroid model system whereby quantitatively monitoring clonal dynamics in two different drug-induced selection environments, namely dabrafenib-resistant 3D-HT-29 and irinotecan-resistant 3D-HCT-116 spheroids. The cellular barcoding technology applied into current model system allowed us to measure frequencies of selection under these environments and hence the presence of both pre-existing and de novo barcode selection, indicating the presence of polyclonal drug resistance. Furthermore, genomic analysis of dabrafenib and irinotecan-resistant spheroids revealed the enrichment of specific SNVs and CNVs for both drug-induced resistances. Overall, our findings provide important insights into drug-induced clonal dynamics in a 3D spheroid model system quantitatively for the first time using cellular barcoding technology.

## Materials and methods

### Cell culture

HT-29 colorectal cancer cell line and its 3D spheroid derivative (3D-HT-29) were maintained in Dulbecco’s Modified Eagle Medium (Biological Industries, Israel) supplemented with 10% Fetal Bovine Serum (Gibco, USA), 1% (v/v) Penicilin-Streptomycin (Biological Industries, Israel) and 1% (v/v) L-Glutamine (Biological Industries). HCT-116 colorectal cell line and its 3D spheroid derivative (3D-HCT-116) were maintained in Roswell Park Memorial Institute‐1640 medium (Biological Industries, Israel) with 10% Fetal Bovine Serum, 1% (v/v) Penicilin-Streptomycin and 1% (v/v) L-Glutamine. Cells were incubated at 37°C in a humidified atmosphere of 5% CO_2_. The mycoplasma negativity in cell lines was routinely confirmed by a PCR-based method [40].

### Cellular barcoding

CloneTracker™ (Cellecta, USA) lentiviral barcode library was used for the barcoding of HT-29 and HCT-116 cell lines. As a first step, lentivirus production was performed on HEK293T cell by co-transfecting the barcode library pool 1 plasmid with pCMV-VSVG (Addgene: 8454) and pCMV-dR8.2 dvpr (Addgene:8455) plasmids using Lipofectamine™ 2000 transfection reagent (Thermo Scientific, USA). Lentiviral infection of HT-29 and HCT-116 cell lines were performed in the presence of 0.8 μg/ml polybrene. The Multiplicity of Infection (M.O.I.) 0.1 was achieved under the selection of puromycin with following concentrations: 1.5 μg/ml for HT-29 and 1 μg/ml for HCT-116.

### 3D spheroid formation

To establish 3D spheroids from barcoded HT-29 and HCT-116 cell lines, poly-HEMA (Santa Cruz, USA), and methylcellulose (Sigma Aldrich, USA) mixture was used [41–43]. Poly-HEMA prevents cells from adhering to the surface and helps them clump together to generate spheroids, while methylcellulose was added medium to obtain evenly spheroid size and avoid over-aggregation. Poly-HEMA was dissolved in 95% ethanol and used at a final concentration of 5 mg/ml to coat a tissue culture dish. Following this, the plates were kept in a sterile hood with lids open for 72 hours. Of note, 5 mg/ml methylcellulose was added additionally to complete growth medium for culturing of 3D spheroids. Methylcellulose added medium was prepared fresh each time and sterilized using a 0.22 μm filter.

### Generation of dabrafenib-resistant 3D-HT-29 and irinotecan-resistant 3D-HCT-116 spheroids

Previously frozen barcoded HT-29 and HCT-116 at 4x10^4^ cell number were thawed. Initially, 1x10^7^ barcoded HT-29 and 1x10^7^ barcoded HCT-116 cells were seeded equally onto poly-HEMA coated 4xT75 flask. Total 4 replicates for each cell line group were designed as 3 drug-treated replicates (A, B and C) and a DMSO control. After seeding, cells were allowed to form spheroids for 3 days in full growth medium containing 5 mg/ml methylcellulose. After the formation of spheroids, barcoded 3D-HT-29 spheroids were treated with dabrafenib for 16 weeks at increasing doses starting from IC50/10 (9 μM) dose until final IC50/2 (45 μM) dose with monthly increase of the dosing, and barcoded 3D-HCT-116 cells were treated with irinotecan for 4 weeks at increasing doses starting from IC50/4 (0.6 μM) dose until final IC50 (2.5 μM) dose with weekly increase of the dosing. Media of the cells were replaced with fresh media containing drug twice a week. For medium change, the spheroids were collected from the medium in the flasks, transferred into 15 ml falcons, and centrifuged at 260 x g for 5 min. Following the centrifugation, the supernatant was discarded and the pellet containing spheroids was dissolved in fresh medium with drug and 5 mg/ml methylcellulose. Finally, dissolved pellet containing spheroids were seeded back to the T75 flasks.

### Barcode sequencing analysis

Barcode sequences composed of 14 bp and 30 bp length variable nucleotides were sequenced with MiSeq Platform (Illumina, Inc.) using 150 bp paired end sequencing. The Illumina MiSeq Platform provided the barcode sequencing results of drug resistant harvested cell populations, their initial and DMSO controls and medium samples FASTQ format. Quality control of FASTQ files were evaluated using the FASTQC tool and reads with Phred scores less than 20 were masked from further analysis. Reads were further trimmed with Trimmomatic (parameters for forward reads: HEADCROP:20 CROP:48 and parameters for reverse reads: HEADCROP:79 CROP:48) to reveal 48 bp long unique barcode sequences after removing 20 bp and 79 bp long constant sequences at tails [44]. One million barcode sequences were re-generated computationally according to the Cellecta barcode library excel file (Cellecta-NGS-QC-CloneTracker-XP-10x1MBarcode3-Lib-RFP.xlsx) and saved as a FASTA file by using the R package "insect". Indexing of detected barcode sequences was carried out using the Salmon tool (parameters: k:47) [45]. The total read number of barcodes in forward and reverse directions was counted and written to an SF file using the Salmon function, which was then converted to a CSV file.

The growth rates of the detected unique barcodes were calculated based on their frequencies in dabrafenib-resistant 3D-HT-29 and irinotecan-resistant 3D-HCT-116 biological replicates (A, B, C) and their DMSO controls by using the below formula [38].


$$r=\frac{1}{T}log(\frac{f_{R}}{f_{0}})$$

In this equation, T represents the time (week) that passes between the first drug treatment until the end of the experiment while fR represents the frequency of the individual barcode(s) in the replicate and f0 represents the frequency of individual barcode(s) in the corresponding DMSO control. Barcode with counts less than 2 in all resistant samples and DMSO control were not included for further analysis. Barcodes were classified into three groups based on their growth rates and number of occurrences in the replicates: barcodes having a positive growth rate and detected in at least two replicates were classified as pre-existing; barcodes having a positive growth rate but detected only in one replicate were classified as de novo and those with negative growth rates were classified as sensitive barcodes [38]

### Whole exome sequencing

Ten whole exome sequencing libraries were prepared from genomic DNA by using Twist Comprehensive Exome kit according to the manufacturer’s instructions. The libraries were pooled and sequenced on the MGI DNBSEQ-G400 platform. As determined by Qualimap tool, the mean (of means) coverage achieved was 226X (min 48X, max 945X) for HT-29 and 183X (min 36X, max 648X) for HCT-116 cell lines (**S1 Table**).

Trimming was performed with trimmomatic and reads with a mean quality value < 20 were discarded. Then reads were aligned to hg38 reference genome by using the Burrows–Wheeler Aligner tool. PCR duplicates were marked using GATK (v4.3.0.0) MarkDuplicatesSpark. Recalibration of quality scores were carried out with the BaseRecalibrator-ApplyBQSRSpark. Somatic mutations were called using Mutect2. Variants were filtered by FilterMutectCalls which was followed by annotation with Funcotator. Only the variants that have a minimum coverage of 10 reads in all samples and a location within the target regions of the exome capture panel were included in further analysis (**S2 and S3 Tables**). For each group, variants exhibiting a 2x enrichment in VAF only in the treated lines (RepA, RepB and RepC) but not in DMSO compared to initial lines were determined (**S4 and S5 Tables**).

Copy Number Alterations (CNA) was detected for each group independently with Control-FREEC (v.11.6) [46] using initial lines as a control and assuming a ploidy of 2 and 3 for HCT-116 and HT-29, respectively. Significant CNAs (p-value < 0.05) which were detected in at least two replicates but not in DMSO were identified and real time RT-PCR candidates were selected among cancer-related genes overlapping to these regions (**S6 and S7 Tables**).

### Dose response curve analysis

To assess the resistance to dabrafenib and irinotecan in 3D-HT-29 and 3D-HCT-116 cells, respectively, cell viability analysis was performed. For this purpose, spheroids from DMSO control groups and 3 replicates (A, B and C) in T75 flasks were collected in 15 ml falcon tubes and centrifuged at 260 x g for 5 minutes. The pellets were treated with 5 ml Trypsin at 37°C for 10 min and dissociated into single cells. Then, 1.5x10^4^ cells were seeded into poly-HEMA coated 96-well plates with medium containing methylcellulose. After allowing 3 days for the cells to form spheroids, 72 hours dabrafenib and irinotecan treatment were performed. Cell Titer-Glo® 3D (Promega, USA) cell viability protocol was used for IC50 determination of all of the spheroids. We used GraphPad Prism software to calculate IC50 values in all cell viability results through following the software’s guidelines [47]. The drug concentrations used in the experiment were initially transformed as a logarithmic concentration. Then, we analysed the data using log(inhibitor) vs. normalised response model and non-linear regression. The dose response model estimated the IC50 value according to the rest of data points used in the regression model.

### Real time RT-PCR analysis

Total RNA was isolated from 3D-HT-29 DMSO control, dabrafenib-resistant 3D-HT-29 Replicates A, B and C; 3D-HCT-116 DMSO control, irinotecan-resistant 3D-HCT-116 Replicates A, B and C cells using the Invitrogen PureLink RNA Mini Kit (Thermo Fisher Scientific, USA) according to manufacturer’s instructions. The concentration and purity of the isolated RNA samples were determined using the NanoDrop instrument (Biodrop, UK). Complementary DNA (cDNA) synthesis was performed by using 1 μg total RNA, 5 μM oligo d(T), 1X ProtoScript II Reaction Mix, and 1X ProtoScript II Enzyme Mix as a final concentration (ProtoScript II First Strand cDNA Synthesis Kit, New England BioLabs, USA).

Real time RT-PCR reaction was performed using GoTaq® qPCR Master Mix (Promega, USA). *GAPDH* gene was used as a housekeeping gene for the analysis. Each reaction was carried out in three biological replicates. Data were displayed as fold changes and analysed using the 2^−ΔΔCt^ method [48]. The list of primers used are as following (**Table 1**).

**Table 1:** List of primers used in real time RT-PCR analysis.

Name	Sequence	Tm
CCNE1 Forward	TTGCTGCTTCGGCCTTGTAT	60°C
CCNE1 Reverse	GCTTTCTTTGCTCGGGCTTT	
NSD3 Forward	CCAGAGAGGGCGTGGGTTCA	63°C
NSD3 Reverse	TCTGAGGTCGGGGTTTCCGA	
RNF6 Forward	CTGTGGCTAGGCGAACAAGA	60°C
RNF6 Reverse	GTGAAGCGTTAGCTCGAGGA	
PRKDC Forward	AGGGAACACCCTTTCCTGGT	60°C
PRKDC Reverse	CTCCTCTTGGGACATGGTGT	
ABCB1 Forward	ACAGAAAGCGAAGCAGTGGT	60°C
ABCB1 Reverse	ATGGTGGTCCGACCTTTTC	
ABCG2 Forward	AGATGGGTTTCCAAGCGTTCAT	60°C
ABCG2 Reverse	CCAGTCCCAGTACGACTGTGACA	
GAPDH Forward	TGGTCACCAGGGCTGCTTTT	62°C
GAPDH Reverse	ACTCCACGACGTACTCAGCG	

### Statistical analysis

For each experiment, at least three biological replicates and a minimum of three technical replicates were performed. Data were examined using t-test and oneway analysis of variance (ANOVA) followed by nonlinear regression tests using GraphPad Prism 8 software (GraphPad Inc.). The results were considered statistically significant at the level of 0.05. All findings were presented as mean ± SD. * = p<0.05, ** = p<0.01, *** = p<0.001, **** = p<0.0001.

## Results

### Cellular barcoding and establishment of 3D spheroids of HT-29 and HCT-116 cell lines

**Fig 1:**
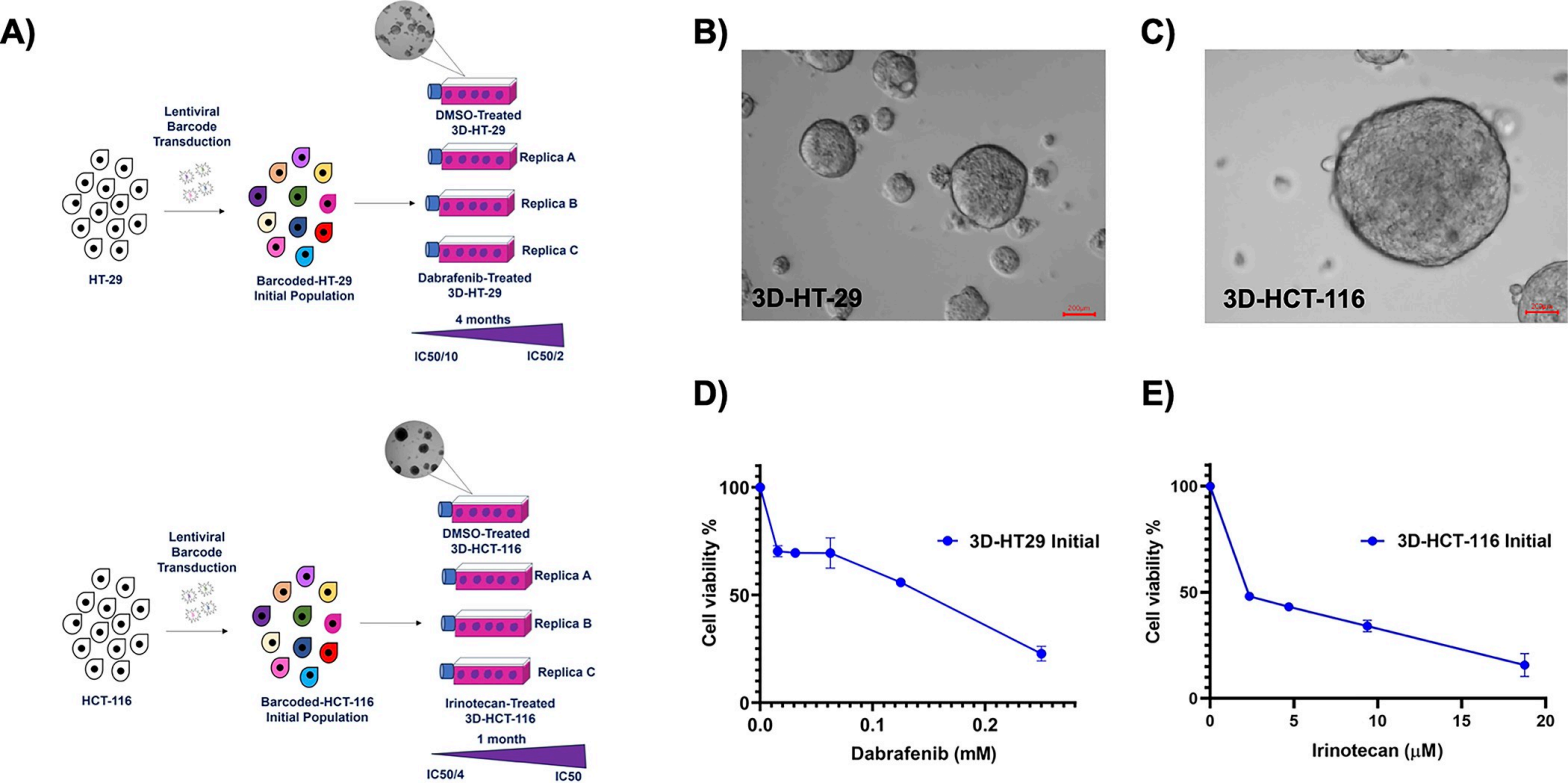
Experimental design for cellular barcoding of 3D spheroids and their establishment. **A.** Schematic of an experimental design. **B.** Bright field microscope image of 3D-HT-29 spheroids and **C.** 3D-HTC-116 spheroids. **D.** The cell viability assay for determining the dabrafenib-dependent sensitives of 3D-HT-29 spheroids and **E.** irinotecan-dependent sensitivities of 3D-HCT-116 spheroids. Error bars represent mean ± SD.

First, utilizing the Cellecta lentiviral barcode library, cellular barcoding of the HT-29 and HCT-116 cell lines was achieved. In the initial HT-29 and HCT-116 cell populations, a multiplicity of infection (M.O.I) of 0.1 was employed to introduce a unique barcode per single cell. This was then followed by the establishment of 3D spheroids of HT-29 (3D-HT-29) and HCT-116 (3D-HCT-116) in a poly-HEMA coated flasks (**Fig 1A**). During in vitro culturing, 3D-HT-29 and 3D-HCT-116 spheroids exhibited full 3D forms and displayed spheroid characteristics (**Fig 1B and 1C**). Morphological characteristics examined under the light microscope showed compact and round morphology for both 3D-HT-29 and 3D-HCT-116 spheroids. Size of the spheroids formed by either cell type exhibited comparable results except only in a few areas where 3D-HCT-116 spheroids bigger in size were observed. Smooth surfaces observed in both spheroids limited to estimate total number of cells within the inner layers of these formations. Despite these observations which were made under the light microscope was informative, using scanning electron microscopy can provide more in-depth understanding to better characterize spheroids morphologically.

Before the establishment of drug-resistant derivatives of barcoded 3D-HT-29 and 3D-HCT-116 cells, we aimed to determine their sensitivities against dabrafenib and irinotecan, respectively. For this purpose, we performed the cell viability assays in barcoded 3D-HT-29 and 3D-HCT-116 cell lines treated with dabrafenib and irinotecan, respectively, with the ultimate aim identifying their half-maximal inhibitory concentrations (IC50). The IC50 values of dabrafenib and irinotecan were identified as 0.09 ± 0.05 mM (mean ± SD) and 2.54 ± 0.04 μM (mean ± SD) in barcoded 3D-HT-29 and 3D-HCT-116 cell lines, respectively (**Fig 1D and 1E**). Thus, we successfully generated 3D spheroids from the HT-29 and HCT-116 cell lines and determined their IC50 concentrations for dabrafenib and irinotecan, respectively.

### Generation of dabrafenib and irinotecan resistant derivatives of barcoded 3D-HT-29 and 3D-HCT-116 cell lines, respectively

**Fig 2:**
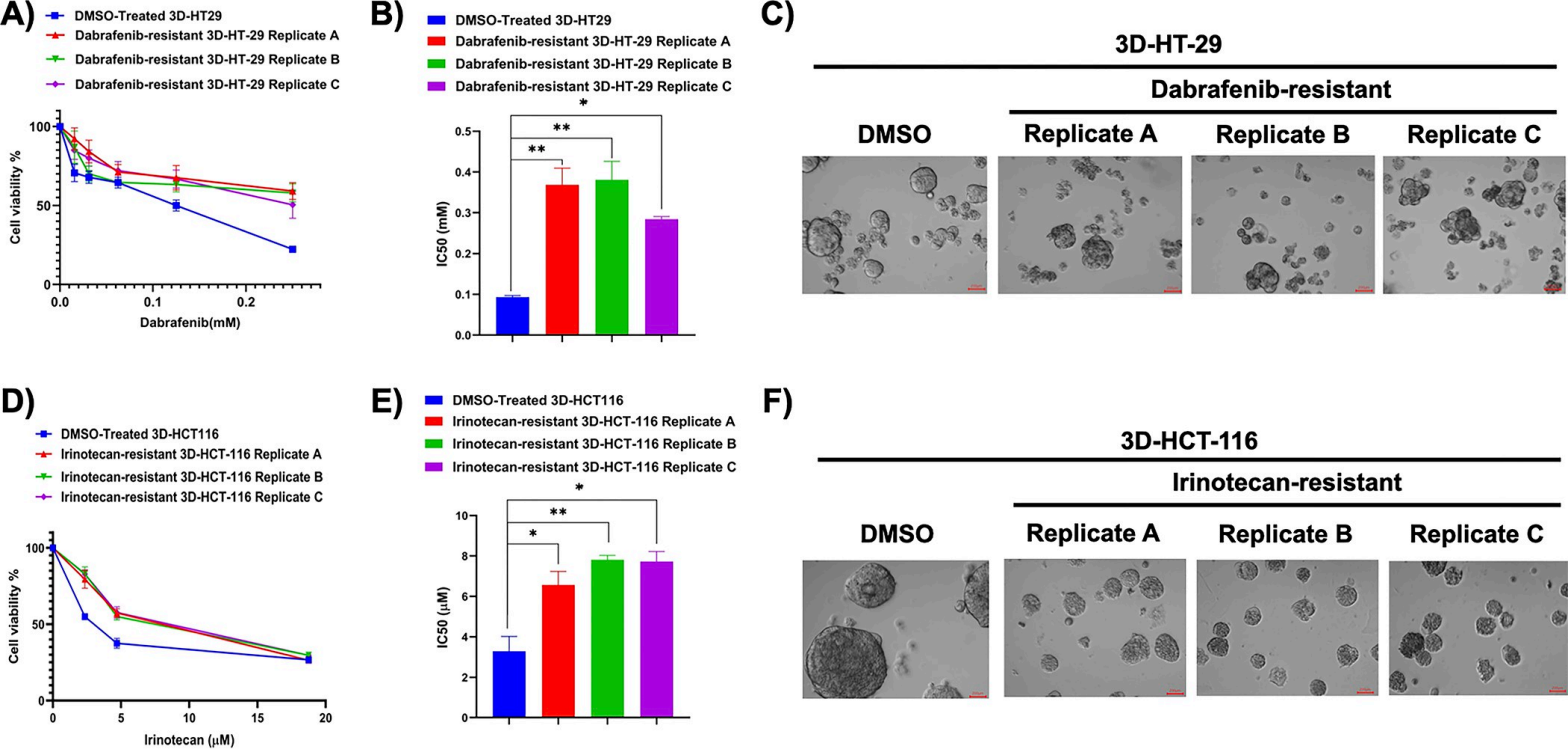
Establishment of drug resistant derivatives of 3D spheroids. **A.** The cell viability assay for the confirmation of dabrafenib resistance in dabrafenib-resistant 3D-HT-29 spheroid replicates A, B and C in comparison to DMSO treated spheroids. **B.** IC50 values of dabrafenib-resistant 3D-HT-29 spheroid replicates A, B and C and DMSO control spheroids are presented using bar chart. The one-way ANOVA test was used for a statistical test. **C.** Brightfield microscope images of dabrafenib-resistant 3D-HT-29 spheroid replicates A, B and C and DMSO control spheroids. **D.** Dose response analysis for the validation of irinotecan resistance in irinotecan resistant 3D-HCT-116 spheroid replicates A, B and C in comparison to DMSO treated spheroids. **E.** IC50 values of irinotecan-resistant 3D-HCT-116 spheroid replicates A, B and C in comparison to DMSO treated spheroids were presented using bar chart. The one-way ANOVA test was used for a statistical test. **F.** Bright field microscope images of irinotecan-resistant 3D-HCT-116 spheroid replicates A, B and C and DMSO treated spheroids are shown. Error bars represent mean ± SD. * = p<0.05, ** = p<0.01.

To establish drug-resistant derivatives of 3D-HT-29 and 3D-HCT-116 cell lines under dabrafenib and irinotecan, these cell lines were treated with respective doses of these drugs. Initial barcoded 3D-HT-29 cells were treated with 9 μM (IC50/10) dabrafenib concentration as a starting concentration and then increased each month for a duration of four months to establish dabrafenib-resistant derivative of 3D-HT-29 cells. Likewise, initial barcoded 3D-HCT-116 cell lines were faced to 0.6 μM (IC50/4) as a starting concentration of irinotecan and this was then followed by increasing the starting concentration weekly for a total of four weeks. Both of these experimental protocols were performed in 3 parallel biological replicates to assess conserved barcodes in the replicates. Specifically, we named the replicates as A, B and C in dabrafenib treated 3D-HT-29 and 3D-HCT-116 groups. In addition, each of these groups had a control DMSO treated 3D-HT-29 and 3D-HCT-116 cell lines. After the treatment of both 3D-HT-29 and 3D-HCT-116 cell line replicates with dabrafenib and irinotecan for respective periods of time, the cell viability assays were performed to assess whether they exhibited increased resistance to these drugs. The cell viability assay showed the detection of increased resistance to dabrafenib in dabrafenib-treated barcoded 3D-HT-29 replicates A, B and C in comparison to barcoded DMSO-treated 3D-HT-29 cell lines (**Fig 2A**). The IC50 values were found as 0.09 ± 0.005 mM, 0.36 ± 0.05 mM, 0.38 ± 0.06 mM, 0.28 ± 0.008 mM (mean ± SD) in DMSO-treated 3D-HT-29, dabrafenib-resistant barcoded 3D-HT-29 replicates A, B and C, respectively. The quantification of increased IC50 values in barcoded 3D-HT-29 replicates A, B and C were found as 4.49-, 4.71-, and 3.25-fold in comparison to barcoded DMSO-treated 3D-HT-29, respectively (**Fig 2B**). Furthermore, acquiring images from dabrafenib-resistant 3D-HT-29 replicates A, B and C as well as DMSO control group showed the morphological similarity across all dabrafenib-resistant replicates with more disorganized structures in comparison to control DMSO treated 3D-HT-29 spheroids, suggesting that dabrafenib resistance may facilitate the observed phenotype (**Fig 2C**). As before, the cell viability assay was performed on irinotecan-treated barcoded 3D-HCT-116 replicates A, B and C which showed increased resistance to irinotecan when compared to barcoded DMSO-treated 3D-HCT-116 cell lines (**Fig 2D**). The IC50 values were found as 3.2 ± 1.0 μM, 6.5 ± 0.9 μM, 7.8 ± 0.3 μM and 7.7 ± 0.7 μM (mean ± SD) in DMSO-treated 3D-HCT-116, irinotecan-resistant barcoded 3D-HCT-116 replicates A, B and C, respectively. Changes in the resistance-fold changes were quantified and 3D-HCT-116 replicates A, B and C were detected as 2.43-, 2.81- and 2.83-fold in comparison to barcoded DMSO-treated 3D-HT-29, respectively (**Fig 2E**). Resistance fold-changes detected in replicates A, B, and C in both cell lines treated with respective drugs were found as nearly similar, indicating that generation of drug resistant derivatives in these replicates were consistent. Last, images acquired from irinotecan-resistant barcoded 3D-HCT-116 replicates A, B and C in comparison to DMSO control 3D-HCT-116 spheroids exhibited smaller spheroids, suggesting a suppressive effect of irinotecan resistance on 3D-HCT-116 spheroids on their growth or formation based on bright-field microscope images (**Fig 2F**). Overall, these results demonstrate the successful generation of dabrafenib and irinotecan-resistant derivatives of 3D-HT-29 and 3D-HCT-116 cell lines in three parallel replicates as well as the morphological characterizations.

### Characterization of drug-induced selection in 3D-HT-29 and 3D-HCT-116 cells via barcode measurement

**Fig 3:**
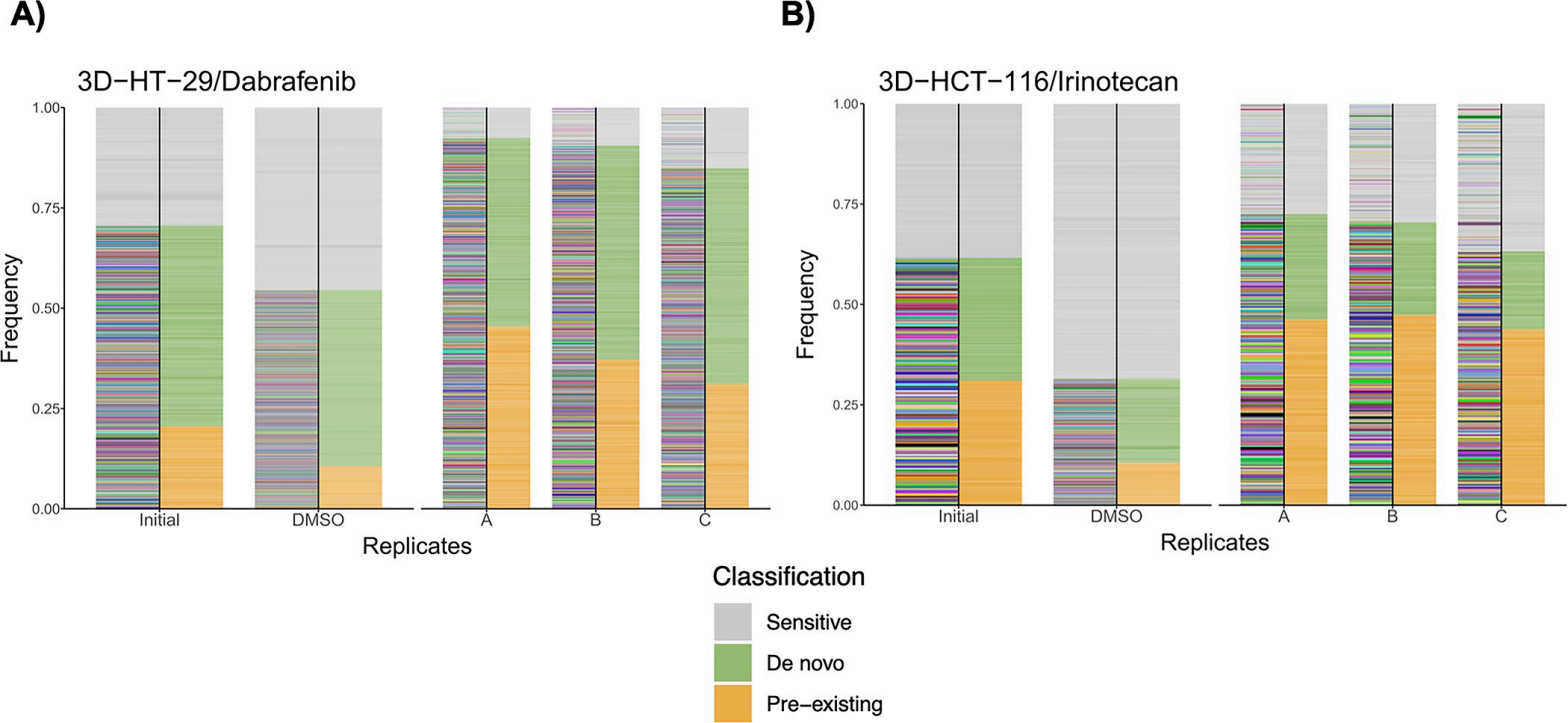
Barcode frequency measurements for the assessment of evolutionary dynamics of drug resistance in 3D spheroids. **A.** Barcode frequency measurements of 3D-HT-29 spheroids namely, initial, DMSO control, dabrafenib-resistant replicates A, B, and C. **B.** Barcode frequency distributions of initial, DMSO control, irinotecan-resistant replicates A, B, and C 3D-HCT-116 spheroids. Barcodes with positive growth rates are classified as pre-existing (amber colour) or de novo (olive green colour) and negative growth rate as sensitive (silver grey colour).

We next aimed to characterize dabrafenib- and irinotecan-induced drug resistance in 3D-HT-29 and 3D-HCT-116 spheroids, respectively. To do so, we leveraged the power of barcoding technology, whereby initial 3D-HT-29 and 3D-HCT-116 cell lines harboured cellular barcodes before their corresponding dabrafenib- and irinotecan-resistant counterparts were established in parallel in three biological replicates, namely A, B, and C. Establishing biological parallel replicates in each drug arm from the same initially barcoded population allowed us to determine whether drug-induced mechanism of resistance was pre-existing or de novo depending on the same barcodes with positive growth rate were detected in biological replicates or not, respectively. In doing so, we performed amplicon based NGS analysis targeting barcode library in dabrafenib-resistant 3D-HT-29 cell line replicates A, B, C, and irinotecan-resistant 3D-HCT-116 cell line replicates A, B, C alongside with their barcoded initial and DMSO-treated groups. Bioinformatic analysis of barcode detection was performed based on identifying barcodes with positive growth rates in three biological replicates both for dabrafenib and irinotecan treated 3D spheroid groups in comparison to DMSO treated groups in each cell line/drug arms. As a result, we found 621, 620, 593 unique pre-existing barcodes in dabrafenib-resistant 3D-HT-29 replicates A, B and C, respectively (**S8 Table**). Moreover, our barcode analysis revealed the detection of 737, 805, 1158 de novo barcodes in dabrafenib-resistant 3D-HT-29 replicates A, B and C, respectively (**S8 Table**). Next, frequency calculations of pre-existing and de novo barcodes were calculated. This analysis demonstrated the detection of pre-existing barcodes (amber colour) as 45.45%, 37.19% and 31.15% and de novo barcodes (olive green colour) as as 46.94%, 53.33% and 53.76% in dabrafenib-resistant 3D-HT-29 replicates A, B and C, respectively (**Fig 3A**). In addition to barcode frequency calculations with positive growth rates for identification of pre-existing and de novo barcodes, sensitive barcodes (silver grey colour) with negative growth rates were also identified as 7.61%, 9.48% and 1.51% in dabrafenib-resistant 3D-HT-29 replicates A, B and C, respectively (**Fig 3A**). We then performed the same barcode frequency calculations in irinotecan-resistant 3D-HCT-116 replicates A, B and C in comparison to DMSO-treated group. Our analysis revealed the detection of 296, 316, 326 unique pre-existing and 198, 167, 206 de novo barcodes in irinotecan-resistant 3D-HCT-116 replicates A, B and C, respectively (**S9 Table**). Furthermore, barcode frequency calculations for irinotecan-resistant 3D-HCT-116 replicates A, B and C exhibited pre-existing barcodes (amber colour) as 46.18%, 47.36% and 43.84% and de novo barcodes (olive green colour) as 26.28%, 23.05% and 19.35%, respectively (**Fig 3B**). Last, sensitive barcodes (silver grey colour) in irinotecan-resistant 3D-HCT-116 replicates A, B and C were found as 27.54%, 29.58% and 36.81%, respectively (**Fig 3B**). Collectively, our findings indicate quantitative measurements of dabrafenib- and irinotecan-induced selection pressures and clonal dynamics as well as the distributions of pre-existing and de novo barcode selection in 3D-HT-29 and 3D-HCT-116 cell lines. Due to the identification of both pre-existing and de novo barcodes detected in both 3D spheroid systems; our results also suggest the presence of polyclonal drug resistance in our experimental system.

### Genomic determinants of drug-induced selection in 3D-HT-29 and 3D-HCT-116 spheroids via whole-exome sequencing

**Fig 4:**
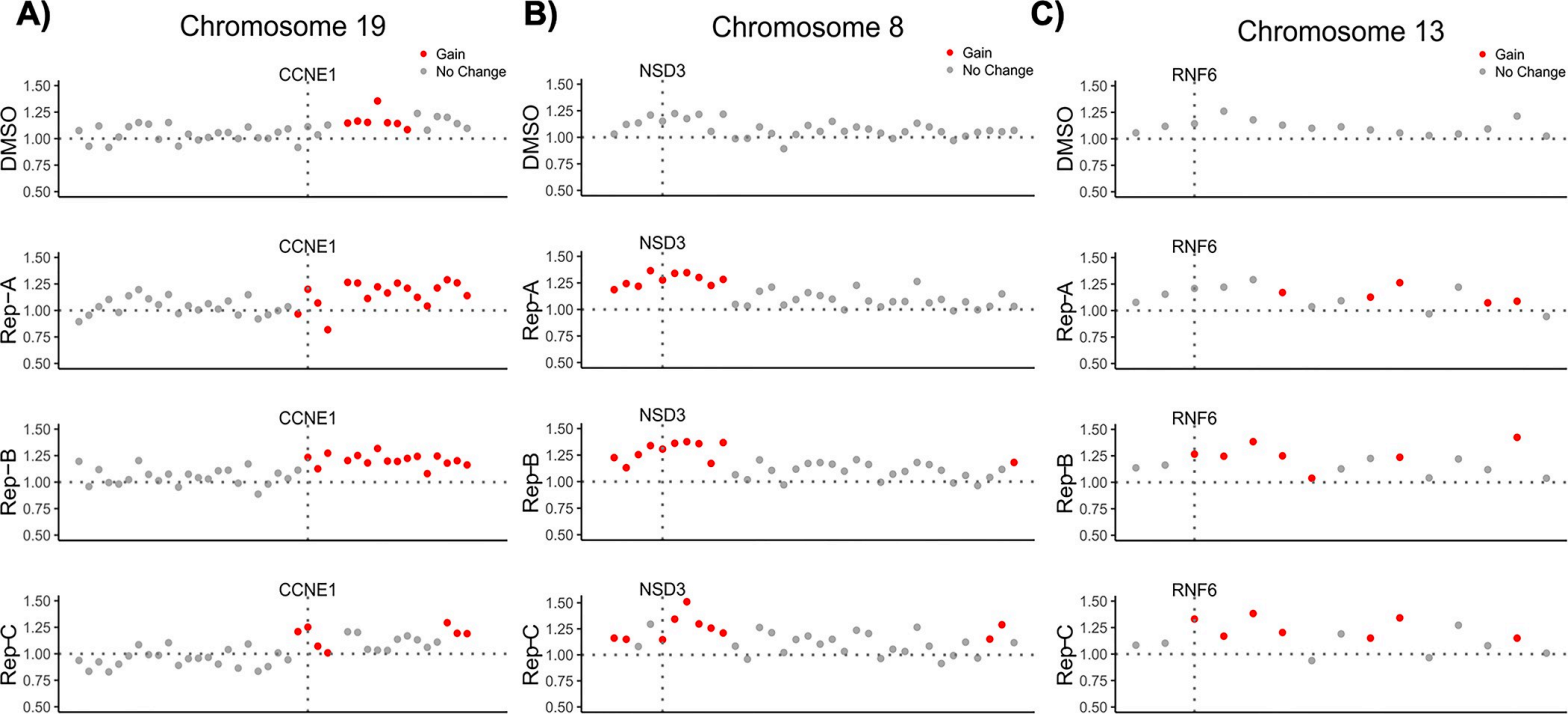
Copy number alterations in dabrafenib-resistant 3D-HT-29 spheroids. Copy number profiling of dabrafenib-resistant 3D-HT-29 replicates A, B and C relative to DMSO control 3D-HT-29 spheroids revealed the presence of a gain in **A.** *CCNE1*. **B.** *NSD3*. **C.** *RNF6* genes.

**Fig 5:**
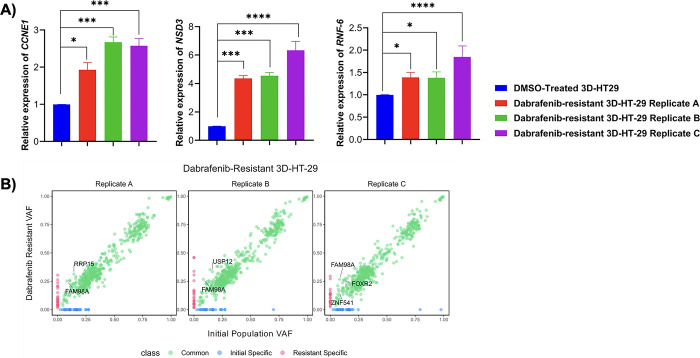
Validation of copy number alterations and detection of somatic single nucleotide variations in dabrafenib-resistant 3D-HT-29 spheroids. **A.** mRNA expression levels of *CCNE1*, *NSD3* and *RNF6* genes were upregulated in dabrafenib-resistant 3D-HT-29 spheroid replicates A, B and C relative to DMSO control 3D-HT-29 spheroid sample. The t-test was used for a statistical test. **B.** Variant Allele Frequency (VAF) analysis of dabrafenib-resistant 3D-HT-29 spheroid replicates A, B and C found enriched SNVs in *FAM98A*, *RRP15*, *USP12* and *FOXR2* genes relative to initial 3D-HT-29 spheroids. Error bars represent mean ± SD. * = p<0.05, *** = p<0.001, **** = p<0.0001.

**Fig 6:**
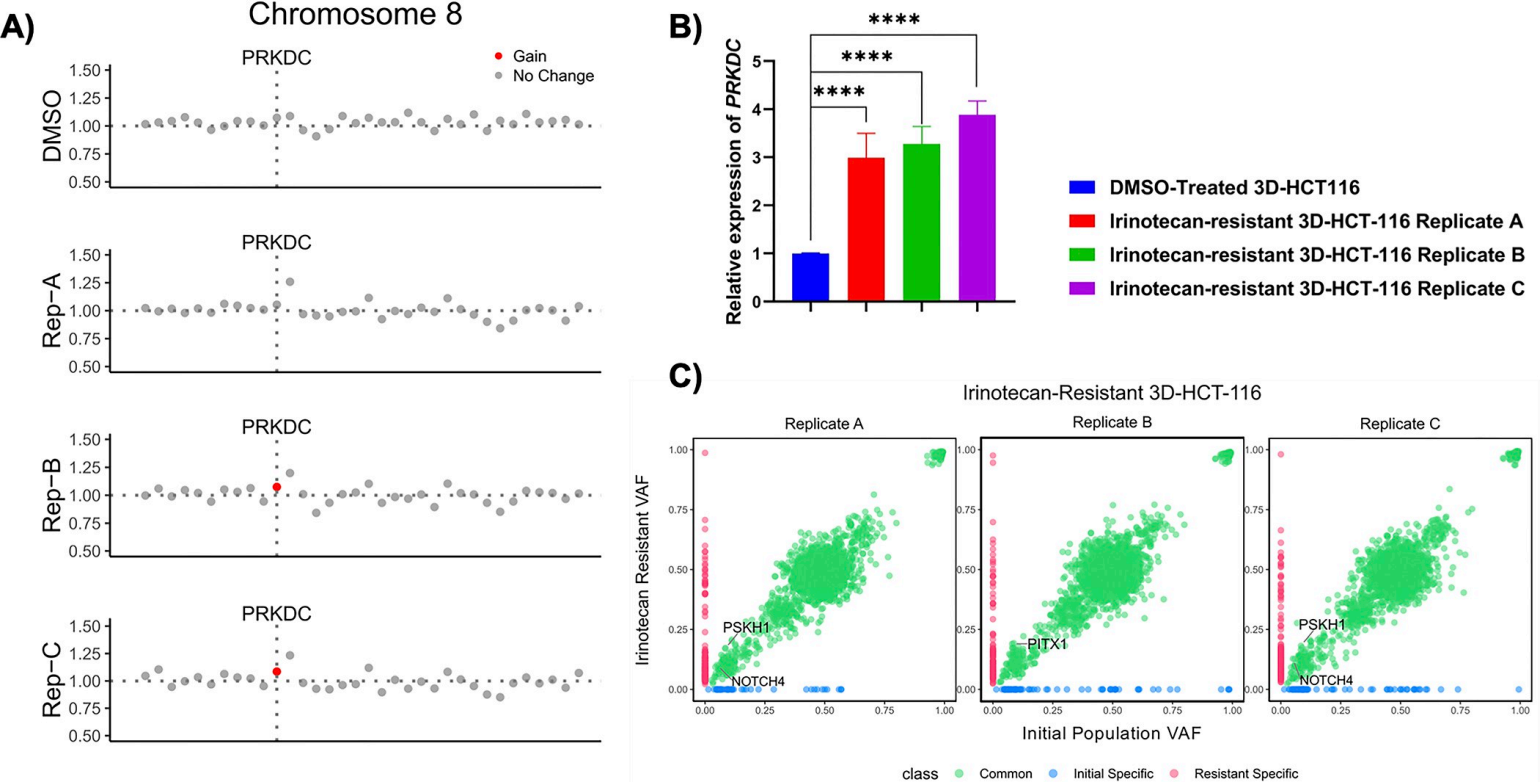
Genomic characterization of irinotecan resistance in 3D-HCT-116 spheroids. **A.** Copy number profiling in all replicates of dabrafenib-resistant 3D-HCT-116 spheroids relative to DMSO control 3D-HCT-116 spheroids showed a gain in the chromosome 8 including *PRKDC*. **B.** *PRKDC* expression levels were upregulated irinotecan-resistant 3D-HCT-116 spheroid replicates A, B and C relative to DMSO control 3D-HCT-116 spheroids. The t-test was used for a statistical test. **C.** The analysis of Variant Allele Frequency (VAF) in irinotecan-resistant 3D-HCT-116 spheroid replicates A, B and C relative to initial 3D-HCT-116 spheroids demonstrated SNVs in *PSKH1*, *NOTCH4*, and *PITX1* were enriched in the replicates. Error bars represent mean ± SD. **** = p<0.0001.

We next wanted to assess possible mechanisms of resistance in dabrafenib-resistant 3D-HT-29 and irinotecan-resistant 3D-HCT-116 spheroids. To do so, we performed whole-exome sequencing (WES) analysis in dabrafenib-resistant 3D-HT-29 replicates A, B, C and DMSO-treated spheroids as well as irinotecan-resistant 3D-HCT-116 replicates A, B, C and DMSO-treated spheroids. Copy-number alteration (CNA) analysis using WES data in dabrafenib-resistant 3D-HT-29 spheroids, detected at least in two replicates, showed focal amplification of *CCNE1*, *NSD3*, *RNF6* genes in comparison to DMSO-treated 3D-HT-29 spheroids (**Fig 4A–4C**) (**S6 Table**). As previously reported, *CCNE1* amplification was detected in colorectal cancer patients and both increased and decreased expression of *CCNE1* were found to be associated with drug resistance in colon cancer, depending on the therapeutics used [49, 50]. Furthermore, the overexpression of *NSD3* was observed in cancer types including colorectal cancer and it enhances the phosphorylation levels of ERK1/2 in CRC cell lines [51, 52]. Last, *RNF6* amplification was frequently observed in CRC, linked to cancer progression and metastasis [53, 54] as well as an increase in the phosphorylation levels of ERK1 in cervical cancer [55]. To assess whether the genomic amplification of *CCNE1*, *NSD3*, *RNF6* genes were reflected in increased mRNA expression, we performed real time RT-PCR. This analysis showed elevated mRNA expression levels of *CCNE1* (0.9-, 1.6-, 1.5-fold), *NSD3* (3.3-, 3.5-, 5.3-fold), and *RNF6* (0.3-, 0.3-, 0.8-fold) in 3D-HT-29 replicates A, B, and C, respectively, in comparison to DMSO-treated 3D-HT-29 spheroids (**Fig 5A**). In addition, using somatic single-nucleotide variations (SNV) analysis from WES data, we detected a total of 779 common somatic SNVs in all of the samples and found missense mutation c.959C>T p.P320L (chr2:33585374:G:A) in *FAM98A* gene conserved in dabrafenib-resistant 3D-HT-29 replicates A, B, and C (**Fig 5B**) (**S4 Table**). Our analysis also demonstrated unique SNVs associated with only certain replicates in dabrafenib-resistant 3D-HT-29 spheroids which were *RRP15* c.435G>T p.M145I (replicate A), *USP12* c.580A>C p.S194R (replicate B), *FOXR2* c.511G>T p.E171* and *ZNF541* c.4G>A p.D2N (replicate C) (**Fig 5B**) **(S4 Table**). *FAM98A* expression was reported to play a role in promoting CRC tumorigenesis [56]. Moreover, high levels of *FAM98A* were detected in 10 out of 12 CRC patients and facilitates resistance to 5-fluoroucail in colorectal cancer by suppressing ferroptosis [57]. Of note, we have not detected amplification of *CCNE1*, *NSD3*, *RNF6* in irinotecan-resistant 3D-HCT-116 replicates A, B, C, suggesting a possible sensitivity of dabrafenib-resistant 3D-HT-29 spheroids for irinotecan. The irinotecan-resistant 3D-HCT-116 replicates B and C showed a weak, but statistically significant, focal amplification in *PRKDC* gene located in chromosome 8 (**Fig 6A**) (**S7 Table**). As a result, we found that *PRKDC* mRNA levels were upregulated as 1.9-, 2.2-, 2.8-fold in 3D-HCT-116 replicates A, B, and C, respectively, in comparison to DMSO-treated 3D-HCT-116 spheroids, respectively (**Fig 6B**). Next, we investigated the presence of SNVs in irinotecan-resistant 3D-HCT-116 replicates A, B and C as a result of irinotecan-induced resistance. In this analysis, we discovered a total of 2567 somatic SNVs in all samples and detected missense mutations in *PSKH1* c.1210C>T p.R404C and *NOTCH4* c.1041G>T p.W347C genes in replicates A, C and in *PITX1* c.580C>T p.L194F gene in replicate B of irinotecan-resistant 3D-HCT-116 spheroids (**Fig 6C**) (**S5 Table**). Elevated *PSKH1* expression was involved in regulating migration and invasion of colon cancer cells [58]. Moreover, increased Notch4 expression is known to facilitate drug resistance in different cancer types including CRC [59, 60]. Overall, our findings suggest possible mechanisms of resistance mediated by CNVs and SNVs in certain genes driving dabrafenib or irinotecan-induced resistance in 3D-HT-29 or 3D-HCT-116 spheroids distinctively as none of the genomic changes detected by our analysis were shared between each drug treatment groups.

### Multidrug resistance in dabrafenib and irinotecan-resistant 3D-HT-29 and 3D-HCT-116 spheroids, respectively

**Fig 7:**
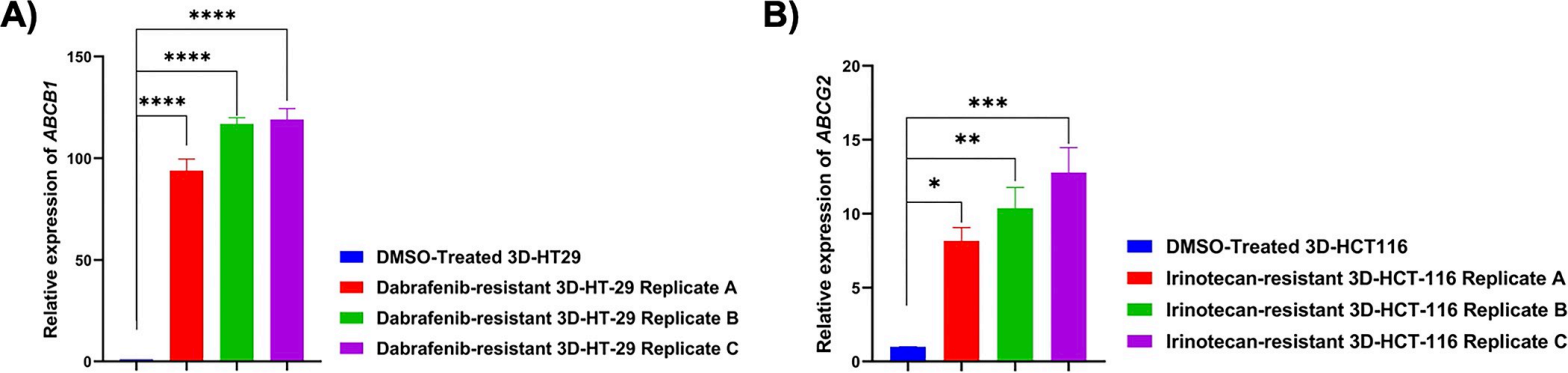
mRNA expression levels of MDR-1 genes. **A.** mRNA expression levels of *ABCB1* was upregulated in dabrafenib-resistant 3D-HT-29 spheroid replicates A, B and C relative to DMSO control 3D-HT-29 spheroid sample. The t-test was used for a statistical test. **B.** *ABCG2* expression levels were upregulated in irinotecan-resistant 3D-HCT-116 spheroid replicates A, B and C relative to DMSO control 3D-HCT-116 spheroids. The t-test was used for a statistical test. Error bars represent mean ± SD. * = p<0.05, ** = p<0.01, *** = p<0.001, **** = p<0.0001.

One of the key reasons for drug resistance is the upregulation of drug efflux pumps, which impairs drug responsiveness and limits therapeutic success [61, 62]. We aimed to assess whether multiple drug resistance (MDR) may be one of the mechanisms of dabrafenib- and irinotecan-induced drug resistance in 3D-HT-29 and 3D-HCT-116 spheroids. To check this, we looked at the mRNA expression levels of two MDR genes namely, *ABCB1* and *ABCG2* in both dabrafenib- and irinotecan-resistant spheroids using real time RT-PCR analysis. ABCB1, also known as P-glycoprotein, is one of the best characterized ABC transporters involved in restricting the efficacy of treatment in CRC and its elevated levels could predict worse prognosis [63, 64]. Moreover, elevated levels of *ABCG2* were correlated with chemotherapy resistance in colon cancer cells [65]. Our analysis showed that *ABCB1* gene was upregulated in dabrafenib-resistant 3D-HT-29 replicates A (92-fold), B (115-fold) and C (118-fold) in comparison to DMSO-treated 3D-HT-29 spheroids (**Fig 7A**), as *ABCB1* a known substrate for dabrafenib [66]. Furthermore, we found that *ABCG2* gene expression levels were upregulated in irinotecan-resistant 3D-HCT-116 replicates A, B, and C in comparison to DMSO-treated 3D-HCT-116 spheroids (**Fig 7B**), alongside with previous findings where *ABCG2* was reported to facilitate irinotecan response [67]. Collectively, our findings demonstrate that dabrafenib and irinotecan-induced resistance mechanisms are separately regulated by upregulation of *ABCB1* and *ABCG2* genes in 3D-HT-29 and 3D-HCT-116 spheroids, respectively.

## Discussion

In this study, we show quantitative measurements of clonal dynamics, via cellular barcoding technology, induced by dabrafenib- and irinotecan-resistance in 3D spheroids generated from HT-29 and HCT-116 cell lines. The cellular barcoding method used in the current 3D spheroid experimental system helped to identify the presence of both pre-existing and de novo barcodes under dabrafenib and irinotecan-mediated selection pressures, exhibiting polyclonal drug resistance. Furthermore, we discovered that dabrafenib and irinotecan-induced drug resistance were mediated by CNVs and SNVs observed in 3D-HT-29 and 3D-HCT-116 spheroids, which helped in understanding the underlying genomic alterations of drug resistance in our 3D-spheroidal experimental system. Overall, our study provides new insights into quantitative measurements and genomic determinants in the 3D-HT-29 and 3D-HCT-116 spheroid systems.

Deciphering drug-induced evolutionary dynamics is key to understanding distinct cellular subpopulations that lead to drug resistance [22]. In many carcinomas, the mechanism of drug resistance, whether pre-existing or emerging de novo, remains a therapeutic challenge [68–70]. Given that 3D spheroid systems closely mimic tumor dynamics [71], our findings could impact on clinical setting via detecting rare pre-existing and de novo-arising resistant cells in 3D-HT-29 and 3D-HCT-116 spheroids in response to dabrafenib or irinotecan pressures. Cellular barcoding technology, with sensitivity down to detecting 1 in 1 million cells, was important in discovering these rare subpopulations since conventional NGS methods can only detect haplotype frequencies in the range of 1–0.1% [38]. These findings emphasize the importance of using more sensitive sequencing technologies in clinical settings to identify rare drug-resistant subpopulations.

In addition to demonstrating rare resistant subpopulations using cellular barcoding technology, whole-exome sequencing found possible SNVs and CNVs mediating the drug resistance. Our CNV analysis found the amplification of Cylin E1 (*CCNE1*) in response to dabrafenib-induced resistance in 3D-HT-29 spheroids, which was corroborated by previous studies where *CCNE1* amplification was detected in most of the solid cancers, including CRC [50], suggesting CDK2 inhibitors can be used as potential second-line agents to reverse dabrafenib resistance in our system [72]. We also found *RNF6* amplification in dabrafenib-resistant 3D-HT-29 spheroids, which is consistent with previous studies promoting CRC progression via aberrant activation of Wnt/β-catenin signalling pathway by inhibiting GSK3β activity [53, 54]; therefore restoring the GSK3β activity might be one of the ways to overcome the resistance observed in our system. Another amplification detected in irinotecan-resistant 3D-HCT-116 spheroids was in the *PRKDC* gene, which encodes the DNA-dependent protein kinase catalytic subunit (DNA-PKcs) protein and plays an important role in non-homologues end joining repair of DNA double strand breaks [73], suggesting that targeting *PRKDC* might induce DNA damage and increase irinotecan sensitivity [74, 75]. Overall, our findings regarding genomic alterations in relation to dabrafenib and irinotecan resistance in spheroids were consistent with previous research in terms of shedding light on drug resistance mechanisms in CRC and, particularly, recapitulating them in the current spheroid system presented in this study.

Despite our goal to develop a quantitative model system that closely mirrors the evolutionary dynamics of drug-induced selection, it has a number of limitations. First, our 3D spheroid model system lacks stromal cell types such as cancer-associated fibroblasts (CAFs) [76], immune cells [77], and endothelial cells [78], which are required to construct a complete tissue and study their role in mediating drug resistance. Second, our 3D spheroids generated from cell lines lack an extracellular matrix (ECM) component [10]; thus, it would be intriguing to develop a 3D culture system using patient cells embedded in an ECM (e.g., Matrigel or Hydrogel) [79] with the ultimate goal of generating drug-resistant derivatives of these patient samples, also known as patient-derived organoids (PDOs) [80]. Finally, our findings rely on an in vitro system with a population size that is further away from the actual tumor volumes of cancer patients. As a result, future research will be required to use a large number of spheroids in the setting of cellular barcoding to measure clonal evolution in cancer patients. In addition to in vitro data presented in this study, in vivo validation of current findings using an animal model as well as investigating the similarities between these two systems will be important. In the clinic, dabrafenib is recommended as 150 mg, 2 times a day for a patient as an oral tablet [81], and while irinotecan is administrated intravenously as 20mg/ml over 90 minutes every week [82]. The concentration used in the patients depends on the cMax which defined as the maximum serum concentration of a drug in the patient. As standard of care recommends, these drugs are repeatedly administrated to the patients (2 times a day and over minutes every week) in a very different cycle than an in vitro study. This may cause an accumulation and leading to increased exposure of a patient to the given drug. Moreover, the cMax concentrations reported as average values and exhibited interpatient variations which can be quite large not only due to patient’s tolerable and metabolic state but also genetic polymorphisms that may be listed as underlying factors [82]. For these reasons, it is hard to mimic patient relevant dosing in an in vitro setting and even the concentration picked based on a patient concentration might not accurately reflect its in vitro use. Despite these limitations, this study is the first to characterize drug-induced evolutionary dynamics in 3D spheroids with the help of cellular barcoding technology, as well as genetic determinants of drug resistance within the same system. Furthermore, the utilization of cellular barcoding technologies coupled with patient-relevant model systems could have implications for addressing drug resistance in the clinic.

## Supporting information

S1 Table Sample coverage information from whole exome sequencing analysis. (XLSX)

S2 Table 3D-HT-29 common single nucleotide variations. (XLSX)

S3 Table 3D-HCT-116 common single nucleotide variations. (XLSX)

S4 Table 3D-HT-29 two times enriched SNV list in each replicate compared to initial. (XLSX)

S5 Table 3D-HCT-116 two times enriched SNV list in each replicate compared to initial. (XLSX)

S6 Table 3D-HT-29 CNV results. (XLSX)

S7 Table 3D-HCT-116 CNV results. (XLSX)

S8 Table 3D-HT-29 barcode frequencies. (DOC)

S9 Table 3D-HCT-116 barcode frequencies. (DOC)
